# Colony Stimulating Factor-1 Receptor Expressing Cells Infiltrating the Cornea Control Corneal Nerve Degeneration in Response to HSV-1 Infection

**DOI:** 10.1167/iovs.17-22159

**Published:** 2017-09

**Authors:** Ana J. Chucair-Elliott, Hem R. Gurung, Meghan M. Carr, Daniel J. J. Carr

**Affiliations:** 1 Department of Ophthalmology, University of Oklahoma Health Sciences Center, Oklahoma City, Oklahoma, United States; 2 Microbiology and Immunology, University of Oklahoma Health Sciences Center, Oklahoma City, Oklahoma, United States

**Keywords:** HSV-1 infection, de-innervation, IL-6, Macrophages

## Abstract

**Purpose:**

Herpes simplex virus type-1 (HSV-1) is a leading cause of neurotrophic keratitis, characterized by decreased or absent corneal sensation due to damage to the sensory corneal innervation. We previously reported the elicited immune response to infection contributes to the mechanism of corneal nerve regression/damage during acute HSV-1 infection. Our aim is to further establish the involvement of infiltrated macrophages in the mechanism of nerve loss upon infection.

**Methods:**

Macrophage Fas-Induced Apoptosis (MAFIA) transgenic C57BL/6 mice were systemically treated with AP20187 dimerizer or vehicle (VEH), and their corneas, lymph nodes, and blood were assessed for CD45^+^CD11b^+^GFP^+^ cell depletion by flow cytometry (FC). Mice were ocularly infected with HSV-1 or left uninfected. At 2, 4, and/or 6 days post infection (PI), corneas were assessed for sensitivity and harvested for FC, nerve structure by immunohistochemistry, viral content by plaque assay, soluble factor content by suspension array, and activation of signaling pathways by Western blot analysis. C57BL6 mice were used to compare to the MAFIA mouse model.

**Results:**

MAFIA mice treated with AP20187 had efficient depletion of CD45^+^CD11b^+^GFP^+^ cells in the tissues analyzed. The reduction of CD45^+^CD11b^+^GFP^+^ cells recruited to the infected corneas of AP20187-treated mice correlated with preservation of corneal nerve structure and function, decreased protein concentration of inflammatory cytokines, and decreased STAT3 activation despite no changes in viral content in the cornea compared to VEH-treated animals.

**Conclusions:**

Our results suggest infiltrated macrophages are early effectors in the nerve regression following HSV-1 infection. We propose the neurodegeneration mechanism involves macrophages, local up-regulation of IL-6, and activation of STAT3.

The cornea, which receives the densest innervation of the body, can be severely damaged due to the development of peripheral neuropathies.^1^ The corneal nerve network consisting mainly of sensory fibers derived from the trigeminal nerve responds to thermal, mechanical, and chemical stimuli by releasing factors that are crucial to the maintenance and homeostasis of the ocular surface.^1,2^ Impairment of the trigeminal corneal innervation is a leading cause of neurotrophic keratitis (NTK), a degenerative disease associated with corneal epithelial breakdown, impairment of healing, and development of ulceration, melting, and perforation.^3^ Regardless of the stage of diagnosis, the hallmark of NTK is decreased or absent corneal sensation.^3,4^ Herpetic viral infections of the cornea, such as herpes simplex virus type-1 (HSV-1), are thought to be a major cause for the development of NTK.^5–7^ HSV-1 enters into a host cell through a multistep process as a result of fusion between the viral envelope and target plasma membrane through the interactions of the HSV-1 encoded glycoproteins gB, gD, gH, and gL with their cognate receptors.^8,9^ Chronic episodes of viral reactivation allow the virus to replicate and usurp axonal transport to travel from the bodies of sensory neurons within the trigeminal ganglia (TG) to the cornea where the infection elicits a local inflammatory response that can lead to herpes stromal keratitis (HSK). HSK is a degenerative and potentially blinding disease characterized by tissue damage, opacity elicited by episodic immune responses,^10–12^ and vascularization of the normally avascular cornea.^13–15^ HSK is associated with the development of NTK. The loss of corneal sensation in HSK patients, often assessed by loss of a corneal blink reflex, is clearly associated with profound loss of the subbasal nerve plexus.^16^

Despite the increasing interest on the research topic of corneal innervation in regards to HSV-1 infection,^17–23^ the mechanisms behind the changes in the distribution and function of corneal nerves as a consequence of viral infection remain to be addressed. Indeed, the identification of target cells, factors, and their downstream signaling pathways has the potential to expand our understanding of a broad spectrum of peripheral neuropathies that develop in inflammatory disease settings (e.g., diabetes mellitus, bacterial infection, and chemical burns). Our published work^17^ using HSV-1 showed corneal nerve regression and loss of function by 6 to 8 days postinfection (PI). Moreover, we recently demonstrated the inflammatory milieu of the infected cornea including the increased concentration of inflammatory cytokines, such as IL-6, and not local viral replication drives nerve degeneration.^18^ In our published paradigm, infected mice treated with a dexamethasone (DEX) ophthalmic solution exhibited preserved corneal nerve structure and sensitivity to mechanical stimuli that correlated with a consistently decreased influx of macrophages (F4/80^+^Gr-1^−^) in the cornea observed as early as 2 days pi.^18^ Similar to our results, recently published work discussed the likelihood of involvement for both early and late inflammatory cells in the loss of corneal sensitivity during HSV-1 infection.^24^ Through their postulated mechanism, the authors suggested that initially resident macrophages may secrete cytokines or other factors that further exacerbate corneal nerve damage, which eventually causes neurodegeneration.^24^

A large body of evidence supports the idea that innate and adaptive (B and T lymphocytes) immune cells play a critical role in neurodegeneration and regeneration in the peripheral nervous system with macrophages as the most notable cell type operating at the injury site.^25^ Nerve–macrophage interactions have been reported following peripheral nerve injury, with macrophages responsible for the phagocytosis of cellular debris, modulation of Schwann cell activities, and secretion of inflammatory cytokines including IL-1, IL-12, and tumor necrosis factor-α (TNF-α).^26–30^ In addition, macrophages are thought to be educated by the local inflamed microenvironment to promote axonal regeneration by releasing a large number of factors including extracellular matrix proteins, growth factors, cytokines, and chemokines.^31–34^ Within the murine cornea, a reported direct physical association between resident tissue macrophages and nerves has been suggested as an indicator of aberrant neuroimmune communication in diseases, such as NTK and peripheral neuropathy.^35^ This observation led us to question whether infiltrating macrophages would establish similar physical and/or functional associations with corneal nerves influencing the events of nerve retraction during the acute phase of HSV-1 infection. Given the above cited role of macrophages in peripheral nerve injury, the association of the macrophage lineage and corneal nerves, and work in the field linking inflammation with corneal nerve degeneration after HSV-1 infection,^18,21,22,24^ we hypothesize a compartment of the innate immune response elicited by HSV-1 infection in the cornea and not the viral replication within the tissue contributes to nerve regression following infection. To address this question, we used a well-established model of macrophage cell depletion, the Macrophage Fas-Induced Apoptosis (MAFIA) transgenic system.^36–38^ Upon systemic treatment with the dimerizer drug AP20187 and subsequent ocular infection with HSV-1, mouse tissues were analyzed to address the impact of the macrophage lineage in nerve regression, and to identify factors and downstream signaling pathways that might be implicated in the neurodegenerative mechanism. Our results consolidate previous evidence on the cross talk between the immune and nervous systems during nerve degeneration in the cornea^18,19,21,22,24^ and suggest a mechanistic axis comprising a corneal influx of macrophages, increased inflammatory cytokines including IL-6, and activation of the gp130/signal transducer and activator of transcription 3 (STAT3) signaling pathway in the process of virus-induced NTK.

## Methods

### Animals

All animal procedures were approved by the University of Oklahoma Health Sciences Center and Dean McGee Eye Institute Institutional Animal Care and Use Committee and performed in adherence to the ARVO Statement for the Use of Animals in Ophthalmic and Vision Research. MAFIA transgenic (stock number 005070; expanded as a colony at the Dean McGee Eye Institute vivarium) and C57BL/6 (wild type [WT]) mice (stock number 000664) were obtained from The Jackson Laboratory (Bar Harbor, ME, USA). Mice were 6 to 8 weeks old at the time of performing experiments. Prior to harvesting tissue, mice were deeply anesthetized with ketamine/xylazine and euthanized by cardiac perfusion with phosphate-buffered saline (PBS).

### Virus and In Vivo Infection

HSV-1 strain McKrae was propagated on HSV-1 susceptible green monkey kidney (Vero) cells and maintained at a stock concentration of 10^9^ plaque forming units (PFUs)/mL. Anesthetized mice were infected by scarification of the corneal surface followed by the application of 3.0 μL PBS containing virus (10^3^ PFU/eye) as previously described.^39^ Controls were performed as previously described.^17^

### Dimerizer Treatment

MAFIA mice were intraperitoneally (IP) injected daily with 40 μg AP20187 dimerizer (B/B homodimerizer) per injection for 5 consecutive days (Clontech Laboratories, Inc., Mountain View, CA, USA; #635058), as previously described.^36,38^ A group of mice received IP injection with the vehicle (VEH) solution (4% ethanol, 10% PEG-400, and 1.7% Tween 80 in sterile water) serving as controls. The day after the last injection, from AP20187- and VEH-treated groups, mice were infected or not with 10^3^ PFU HSV-1 as described above.

### DEX Treatment

Topical delivery of DEX was performed as previously described.^18,19^ Starting at 2 hours PI, nonanesthetized mice were held by the scruff of the neck, and topically delivered a drop of 0.1% DEX ophthalmic solution (Bausch+Lomb, Inc., Tampa, FL, USA; #001-000050-00) onto their corneas. Control treatments (VEH) were done by applying lubricant eye drops (Allergan, Inc., Irvine, CA, USA) onto each cornea. The treatments were applied four times a day, for up to 8 days pi.

### Corneal Sensitivity

As previously published,^17–19,40^ a Cochet-Bonnet esthesiometer (Luneau *SAS*, Prunay le Gillon, France; #8630-1490-29) was used to test for corneal sensitivity. Briefly, at different times PI, nonanesthetized mice were held by the scruff of the neck and presented a monofilament at lengths ranging from 6.0 to 0.5 cm to elicit a blink response. At each length, the monofilament touched the cornea four times making perpendicular contact with the surface before considering a response negative (no blink response).The lack of blink reflex at a monofilament length of 0.5 cm was recorded as 0. All measurements were performed by the same examiner.

### Viral Plaque Assay

At indicated time points, cornea (both corneas per animal pooled together) or TG (both branches of TG per animal pooled together) samples were homogenized with a tissue miser, clarified by centrifugation (10,000*g* for 1.5 minutes), and then serially diluted onto a confluent lawn of Vero cells in RPMI1640 media (Invitrogen, Life Sciences, Grand Island, NY, USA) containing 10% fetal bovine serum (FBS) and antibiotic/antimycotic reagents. Plaques were visualized and enumerated with the aid of an inverted microscope (Invertoskop, Zeiss, Thornwood, NY, USA) 28 to 32 hours later and quantified as mean log PFU per cornea or log PFU per TG samples as previously described.^19,41^

### Flow Cytometry

Harvested tissues were enzymatically and/or mechanically dissociated in RPMI1640 media containing 10% FBS and antibiotic/antimycotic reagent following established protocols. Corneas were digested in 2.0 mg/mL type 1 collagenase at 37°C (a pair of corneas/sample).^18,19^ Mandibular lymph node (MLN) samples were macerated, and TG samples were mechanically dissociated using a Dounce homogenizer to obtain single-cell suspensions that were filtered through a 40-μm mesh.^19,42,43^ Blood samples (100 μL) were collected from the facial vein of mice using a 4-mm sterile Goldenrod animal lancet (MEDIpoint, Inc., Mineola, NY, USA),^19^ mixed with 5 μL 0.5 M EDTA to prevent coagulation, and treated with 1.0 mL red cell lysing buffer (150 mM ammonium chloride, 10 mM potassium bicarbonate, and 0.1 mM EDTA), as previously descibed.^43^ The single-cell suspensions from each tissue were immunostained for flow cytometric analysis. The myeloid population was broadly defined as CD45^+^CD11b^+^. A subsequent positive selection for GFP^+^ cells was made to select for CD45^+^CD11b^+^GFP^+^ cells (CSF-1R^+^ macrophages/monocytes/dendritic cells [DCs] for MAFIA mice). The antibodies included anti-mouse CD45: eFluor450 (clone 30-F11) and anti-mouse CD11b: PE-Cy7 (clone M1/70) (eBioscience, San Diego, CA, USA). Samples were analyzed using a MacsQuant flow cytometer and MacsQuantify software (Miltenyi Biotec, Bergisch Gladbach, Germany).^44^

### Immunochemistry and Imaging

For immunostaining of cornea flat mounts, eyes were removed and an incision was made posterior to the limbus to dissect the corneas including a margin of sclera. The corneas were fixed, permeated, and incubated overnight sequentially with blocking, primary, and secondary antibody solutions as previously described.^17^ The primary antibodies included were anti-β III tubulin (Abcam, Cambridge, MA, USA; #18207, 1:1000) and anti-CD31 (EMD Millipore, Billerica, MA, USA; #MAB1398Z, 1:100). Negative controls without primary antibodies and using blocking solution (10% normal donkey serum in 0.1% Triton X-100 PBS solution) and subsequently incubated with fluorescently labeled secondary antibodies were performed on cornea frozen sections and showed no positive signal for β III tubulin or CD45 signal. Incisions were made in each sample in order to obtain whole mounts (four quadrants) prior to mounting in 50% glycerol. Imaging of cornea samples was performed on an Olympus FluoView confocal laser scanning microscope (FV500 v5.0 or FV1200; Olympus, Center Valley, PA, USA). Microscope and software settings were identical for all samples within experiments. The size of the Z-stack generated for cornea flatmounts was 12 slices thick (4.77-μm step size) at 10× magnification. To quantify changes in corneal innervation and vasculature, the MetaMorph offline software (version 7.7.0.0; Molecular Devices, LLC, Sunnyvale, CA, USA) was used to calculate the percent threshold area positive for β III tubulin or CD31 staining on acquired confocal images. This threshold area is defined as the percentage of β III tubulin^+^ or CD31^+^ pixels divided by the total number of pixels in the entire image. For each cornea, a representative image from each quadrant was used for analysis (four images per sample where the visual field included the peripheral limbus toward the center of the cornea proper).^17^ For immunostaining of sagittal frozen sections, fixed cornea samples were cryoprotected by overnight incubation with 30% sucrose and then frozen in optimal cutting temperature media. Corneal cryostat sections (16-μm thick) were permeated, blocked, and immunostained with anti-β III tubulin (1:1000) and CD45 (BD Pharmingen, San Jose, CA, USA; #550539, 1:200) antibodies.^17^ Confocal imaging of representative areas was performed to generate a Z-stack of eight slices (2.4-μm step size) at 40× magnification and 1.5× zoom.

### Protein Extraction, Suspension Array, and Western Blot

Corneas and TGs were harvested and homogenized in T-PER tissue protein extraction reagent (Thermo Scientific, Pittsburgh, PA, USA; #78510) supplemented with 1× protease inhibitor cocktail (EMD Millipore; #539131) and 1× sodium orthovanadate (Santa Cruz Biotechnology, Dallas, TX, USA; #sc-24948) using a tissue miser (corneas) or a tissue sonicator (TGs). The supernatants from tissue homogenates were assayed for protein content using a BCA protein method (Thermo Scientific; #23225). Suspension array analyte concentrations were determined using a Bio-Rad Bioplex system (Bio-Rad Laboratories, Hercules, CA, USA). Milliplex Map Luminex-based assays were used to quantify inflammatory cytokines (IL 1-α, IL-1β, IL-6, and IFN-γ) (EMD Millipore; #MCYTOMAG-70K) and proangiogenic factors (angiopoietin [ANGPT]-2, fibroblast growth factor [FGF]-2, and hepatocyte growth factor [HGF]) (EMD Millipore; #MAGPMAG-24K).^18,45^ The concentration of each detected analyte was normalized to the protein content in the sample, and results were expressed as pg analyte/mg protein. Western blot analysis was performed as previously described.^17,18^ Equal amounts of protein per sample (10 μg) were resolved on SDS 4-20% gradient polyacrylamide gels (Thermo Scientific, #EC60285) prior to transferring onto nitrocellulose membranes. Proteins were detected using the following primary antibodies: anti-STAT3 antibody to total STAT3 (Cell Signaling Technology, Danvers, MA, USA; #4904, 1:2000), anti-phospho-STAT3 to phosphorylated STAT3 (pSTAT3) (Cell Signaling Technology; #9145, 1:1000), anti-p44/42 MAPK (ERK1/2) antibody to total extracellular signal regulated kinase (ERK) (Cell Signaling Technology; #9102, 1:1000), anti-phospho-p44/42 MAPK (ERK1/2) antibody to phosphorylated ERK (pERK) (Cell Signaling Technology; #9101, 1:1000), and anti-β actin (Abcam; #ab6276, 1:10,000). For re-probing blots, membranes were stripped (Thermo Scientific; #46430) prior to blocking and incubation with a different primary antibody. For secondary chemiluminescence detection, blots were incubated with corresponding horseradish peroxidase (HRP)-conjugated secondary antibodies (GE Healthcare Bio-Sciences, Pittsburgh, PA, USA). Imaging of the blots and analysis of band intensities were performed by conventional image analysis using a Kodak in vivo imaging system F Pro and Carestream MI SE 4.4 SE version software (Carestream Health, Inc., Rochester, NY, USA).

### Statistical Analysis

Statistical analysis was performed by using GraphPad Prism 5.0 software (GraphPad software, San Diego, CA, USA). The data are expressed as the mean ± SEM for each group. The unpaired *T*-test comparison was performed to assess the significant differences between two groups. For multiple comparisons, 1-way analysis of variance (ANOVA) was performed followed by the Bonferroni post hoc test.

## Results

### The MAFIA Transgenic Mouse as a Model to Ablate the CSF-1R^+^ Cell Compartment

**Figure 1:**
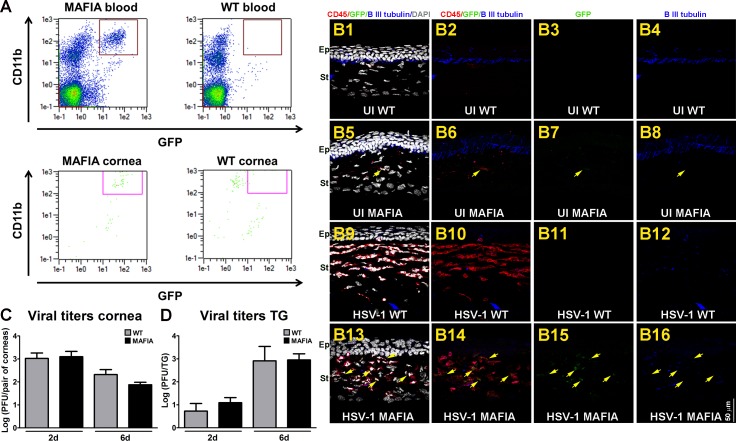
Comparison between MAFIA and WT mouse systems. (A) Representative flow cytometry plots of blood and cornea tissues from UI MAFIA and WT mice. Based on a gating strategy that selects CD45^+^CD11b^+^GFP^+^ cells (cells inside the box), the expected phenotype was found in MAFIA (left) but not in the WT (right) tissues. (B1–B16) Representative confocal images of sagittal frozen sections of corneas at 6 days PI co-stained with CD45 (red) and β III tubulin (blue) antibodies. UI WT corneas showed scarce CD45^+^GFP^−^ cells throughout the stroma. β lIII tubulin^+^ subbasal nerves penetrating the layers of epithelial cells were observed (B1–B4). In the UI MAFIA corneas, the few CD45^+^ cells co-localized with GFP^+^ signal (yellow arrow) and correlated with intact β lIII tubulin^+^ subbasal nerves penetrating the epithelium (B5–B8). Areas of loss of subbasal corneal innervation were observed at 6 days PI, consistent with increased influx of CD45^+^ cells that were GFP^−^ in WT (B9–B12) and co-localized with GFP^+^ signal in the MAFIA (B13–B16) corneas (yellow arrows). DAPI, nuclei counterstained (gray); Ep, corneal epithelium; St, corneal stroma (n = 3–4 corneas per group). (C) Viral content in corneas from WT and MAFIA mice at 2 and 6 days PI as assessed by viral plaque assay and expressed as log PFU/cornea pair ± SEM (n = 5–6 from two independent experiments per time point). (D) Viral content in TG tissue from WT and MAFIA mice at 2 and 6 days PI as assessed by viral plaque assay and expressed as log PFU/cornea pair ± SEM (n = 5–6 from two independent experiments per group) and analyzed by unpaired T-test comparison.

We previously reported the regression of sensory nerves in the cornea after HSV-1 infection was triggered by an immune system-mediated mechanism.^17–19^ Such neurodegeneration could be blocked by topical applications of DEX, a clinically prescribed, anti-inflammatory reagent,^46,47^ onto the HSV-1 infected corneas.^18^ The reported phenotype of corneal nerve structure and function in the presence of DEX therapy was associated with a reduction in macrophage (F4/80^+^Gr-1^−^) influx and was lost when the anti-inflammatory treatment was delayed for 2 days after infection. These findings supported the hypothesis that early events in the inflammatory response to HSV-1 infection, innate in nature (e.g., macrophages), are key in driving corneal nerve loss.^18^ To further study this idea, the MAFIA transgenic mouse model, an established system of conditional depletion of macrophages, was used.^36–38,48,49^ Under the control of the c-fms promoter that regulates the expression of the colony stimulating factor-1 receptor (CSF-1R), MAFIA mice express the fluorescent protein eGFP and a membrane-bound suicide protein. The covalently linked dimerizer AP20187 links the FK506 binding region of the suicide protein and induces caspase-8-dependent apoptosis.^36,37,50^ Prior to applying this mouse system for macrophage depletion, we conducted experiments to validate its suitability for the study of corneal nerves upon HSV-1 infection (Fig. 1). As expected, the populations of monocytes in blood and resident macrophages/DCs in cornea tissue of MAFIA mice were CD11b^+^GFP^+^, whereas the resident mononuclear cells of WT mice did not express GFP (Fig. 1A). Immunohistochemistry (IHC) analysis of sagittal frozen sections stained with CD45 (marker for leukocytes; red signal) and β III tubulin antibodies (pan neuronal marker; blue signal) confirmed this phenotype as well (Figs. 1B1–B16). Naive corneas from uninfected (UI) WT and MAFIA mice possessed scarce CD45^+^ cells throughout the stroma that were GFP^−^ (Figs. 1B1–B3) and GFP^+^, respectively (Figs. 1B5–B7). This differential phenotype was more robust 6 days PI with HSV-1, when the increased leukocyte infiltrate in the cornea of mice co-localized with GFP expression in the MAFIA but not in the WT mice (Figs. 1B9–B11 versus B13–B15). Both WT and MAFIA UI, naive corneas possessed intact subbasal innervation that penetrated the epithelial cell layers **(**Figs. 1B4 versus B8) and was extensively lost at 6 days PI (Figs. 1B12 versus B16). The replication and spread of HSV-1 in the cornea and TG of MAFIA mice were similar to those found for the WT control mice at 2 and 6 days PI **(**Figs. 1C, 1D).

**Figure 2:**
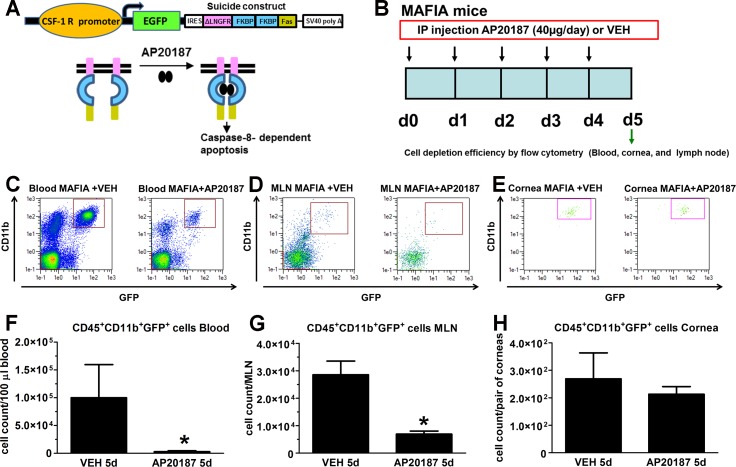
Effect of dimerizer treatment on the CSF-1R^+^ cell population of MAFIA mice. (A) Representation of suicide construct constitutively expressed under the CSF-1 R promoter in MAFIA mice (Burnett et al.^36^) along with the principle of cell depletion upon treatment with the dimerizer AP20187 (based on illustration by Aikawa et al.^50^). (B) Representation of paradigm followed to achieve ablation of CSF-1 R expressing cells. MAFIA mice were IP injected daily with 40 μg AP20187 per injection for 5 days or with the VEH. The day after cessation of treatment, blood, MLN, and cornea tissues were harvested from UI mice and processed for assessment of cell depletion by flow cytometry. (C–E) Representative flow cytometry plots compare CD45^+^CD11b^+^GFP^+^ cell populations from blood (C), MLN (D), and cornea (E) of MAFIA mice after treatment. After the fifth injection of AP20187, a significant decrease in the macrophage/dendritic cell population was found in blood and lymph node but not in the cornea tissue. (F–H) Bar graphs for each tissue summarize the mean phenotypic leukocyte count ± SEM (n = 4–8 per group in F and n = 3 per group in G, H), *P < 0.05 by unpaired T-test comparison.

To study cell depletion efficiency of the systemic dimerizer treatment, MAFIA mice were treated for 5 consecutive days with AP20187 or VEH as described under Methods (principle of depletion illustration in Fig. 2A, modified from Aikawa et al.^50^). Twenty-four hours after cessation of treatment, blood, MLN, and cornea tissues were harvested for flow cytometric analysis of CSF-1R^+^ cells, based on CD45, CD11b, and GFP expression (Fig. 2B). In comparison to MAFIA mice treated with VEH, the distinct population associated with the monocyte/macrophage lineage was significantly reduced in blood (Figs. 2C, 2F) and MLN (Figs. 2D, 2G), while depletion of the resident population of macrophages/DCs in the cornea was not significant in the dimerizer-treated MAFIA mice (Figs. 2E–H). We interpret these results to suggest while effective in depleting cells in the periphery, in the absence of vessels invading the UI cornea, the dimerizer did not reach the corneal stroma such that the resident population of macrophages and conventional DCs (cDCs) was preserved.

**Figure 3:**
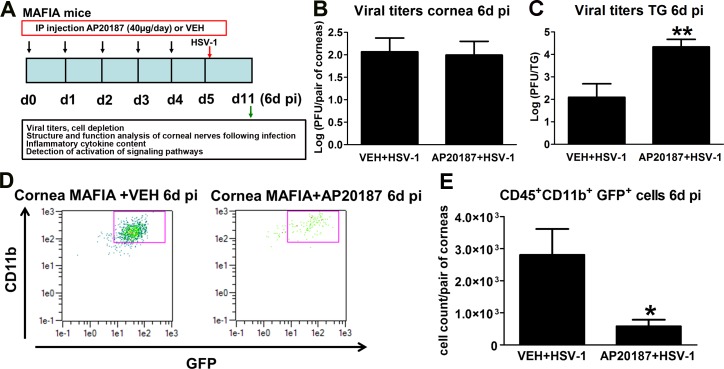
Effect of dimerizer treatment on the CSF-1R^+^ cell population in MAFIA mice following HSV-1 infection. (A) Summary of use of MAFIA mouse model. On the day after the last AP20187 dimerizer/VEH IP injection, mice were infected with 10^3^ PFU HSV-1/cornea and tissues harvested for viral titers or flow cytometry analyses at 6 days PI. (B, C) Viral content in the cornea and TG tissues of MAFIA mice treated with VEH or AP20187 and subsequently infected (at 6 days PI). Bars show mean ± SEM of viral contents in corneas (B) and TG (C) at 6 days PI by plaque assay (n = 5–6 per group from two independent experiments by unpaired T-test comparison). (D) Representative flow cytometry plots compare CD45^+^CD11b^+^GFP^+^ cell populations between VEH and AP20187-treated mice subsequently infected with HSV-1 (at 6 days PI). AP20187-treated mice show a reduction in CD45^+^CD11b^+^GFP^+^ cells infiltrating the infected cornea compared to the VEH-treated counterparts. (E) Bars summarize the mean cell count ± SEM (n = 5 per group, from two independent experiments), *P < 0.05 by unpaired T-test comparison.

Considering the observed successful depletion of CD45^+^CD11b^+^GFP^+^ cells in blood while no significant effect on the resident CD45^+^CD11b^+^GFP^+^ cell population in the cornea following AP20187 treatment (Fig. 2), we questioned whether the MAFIA model subjected to AP20187 treatment and subsequently infected with HSV-1 would be useful in determining the role of infiltrating macrophages, derived mostly from circulating blood, in the neurodegeneration of the infected cornea. To address this question, and to assess viral spread under this treatment, AP20187- and VEH-treated MAFIA mice were ocularly infected with HSV-1. Six days PI, a time point consistent with the physical loss and function of corneal nerves,^17^ corneas and TGs were harvested (Fig. 3A). Data revealed that while AP20187- and VEH-treated mice had similar corneal viral content (Fig. 3B), the TG of AP20187-treated mice had significantly higher viral titers than the VEH-treated group (Fig. 3C). At 6 days PI, the analysis of cell depletion showed a significant decrease of infiltrating macrophages in the cornea tissue of mice treated with AP20187 in comparison to their controls (Figs. 3D, 3E).

### Depletion of the CD11b^+^CSF-1R^+^ Cells Correlates With Preservation of Corneal Nerve Structure and Function Following HSV-1 Infection

**Figure 4:**
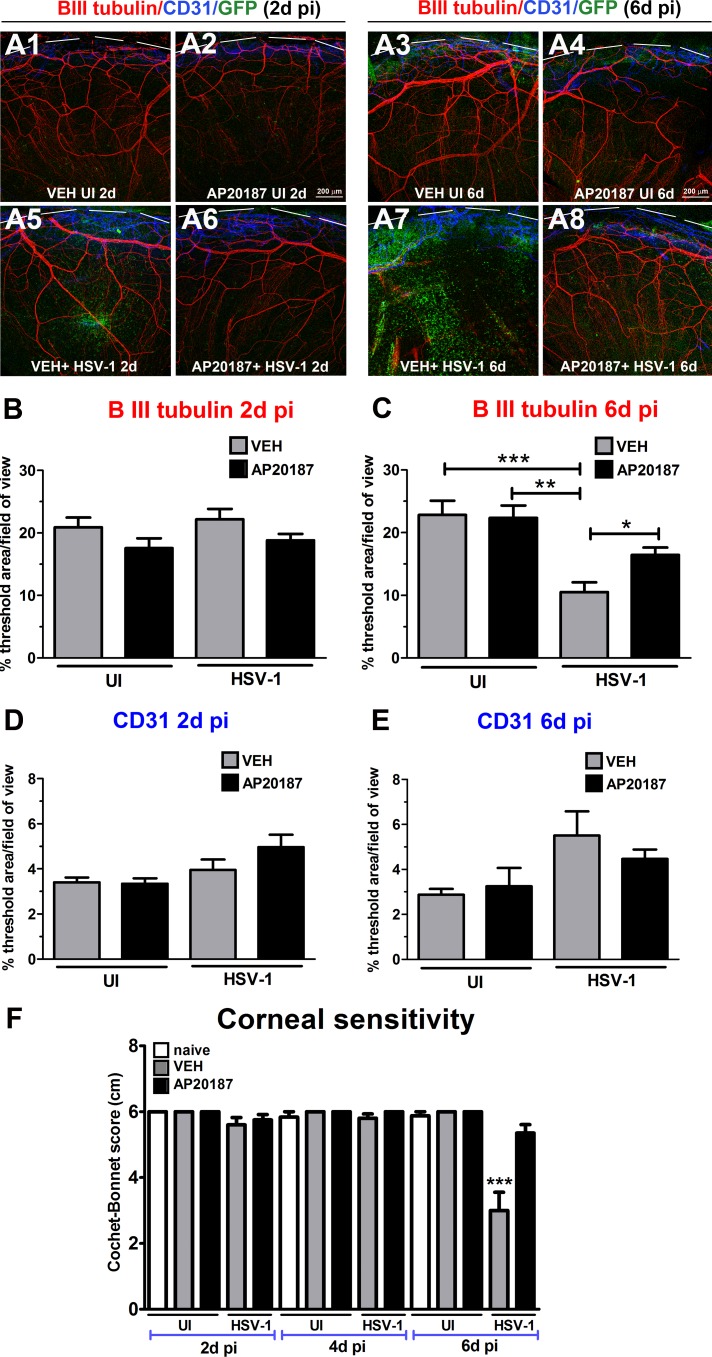
Depletion of CSF-1R^+^ cells correlates with preservation of corneal nerve structure and function following HSV-1 infection. MAFIA mice were treated with daily IP injections of AP20187 dimerizer or VEH. Following the treatment, mice were infected with 10^3^ PFU HSV-1/cornea or left UI as controls and their corneas harvested at 2 or 6 days PI for IHC assessment of corneal innervation and vasculature. (A1–A8) Representative confocal microscopy images of flat mount cornea preparations from UI and infected mice (at 2 and 6 days PI) previously treated with VEH or AP20187. Green: GFP^+^ cells (macrophages/monocytes/DCs); red: nerves (β III tubulin); blue: vessels (CD31); top discontinued white lines depict the limbus margins. Analysis of corneal innervation (B, C) and vascularization (D, E) at the indicated time points PI expressed as mean % threshold area positive for β III tubulin signal per field of view ± SEM and mean % threshold area positive for CD31 signal per field of view ± SEM, respectively. (B–D) n = 6 to 8 per infected group and n = 3 to 4 per UI group from two independent experiments; (C) n = 14 to 16 per infected group and n = 4 to 6 per UI group from three independent experiments; (E) n = 5 to 12 per infected group and n = 4 to 5 per UI group from two independent experiments. (F) Corneal sensitivity was assessed at 2, 4, and 6 days PI, prior to tissue collection. Bars depict mean Cochet-Bonnet score ± SEM for each group (n = 8–10 per infected group and n = 4–6 per UI group at 2 and 4 days PI from two independent experiments; n = 17–18 per infected group and n = 8–10 per UI group at 6 days PI from three independent experiments). (B–F) ***P < 0.001, **P < 0.01, and *P < 0.05 by ANOVA followed by Bonferroni multiple comparison test.

The impact of a decreased influx of macrophages on the innervation and onset of vascularization of the HSV-1 infected cornea was studied by IHC using antibodies against β III tubulin (pan neuronal marker) and CD31 (endothelial cell marker). As previously described for UI corneas,^17^ the UI mice treated with AP20187 or VEH displayed a stromal network formed by thick nerve trunks that ramified into smaller and more superficial branches as they progressed from the periphery toward the center of the cornea (Figs. 4A1–A4). A subbasal network composed by thinner, hairpin-like nerves that projected centripetally and presented a roughly parallel orientation from one another terminating in free nerve endings was observed. In the UI corneas, the presence of resident macrophages/cDCs was traced by the green fluorescence protein signal associated to CSF-1R expression, found sparsely throughout the stroma and on occasion associated with nerve structures, with no apparent differences between treatments (Figs. 4A1–A4). At 2 days PI, HSV-1 infection resulted in areas of increased infiltration of GFP^+^ cells in the corneas of VEH-treated mice (consistent with early increased influx of macrophages and inflammatory monocytes into the infected cornea)^18^ that did not correlate with significant nerve regression or initial vascularization of the tissue. Mice that received AP20187 prior to infection displayed few GFP^+^ cells in the cornea with no significant differences in nerves and vessels in comparison to UI and VEH+HSV-1 conditions at 2 days PI (Figs. 4A5, 4A6, 4B, 4D). By 6 days PI, VEH-treated, HSV-1 infected corneas presented with larger areas of GFP^+^ cell infiltrate than at 2 days PI. The cellular infiltrate was associated with areas of extensive and significant nerve loss (thick stromal and fine subbasal plexus of nerves) compared to UI corneas. AP20187- treated MAFIA mice showed no significant increase in the GFP^+^ cell infiltrate in the cornea associated with a significant preservation of the structure of stromal and subbasal nerves at 6 days PI compared to the VEH-treated, MAFIA mice. Analysis of blood vessels showed no differences between groups at this time point (Figs. 4A7, 4A8, 4C, 4E). Compared to UI corneas (naive, or treated with VEH or AP20187) and infected corneas at 2 to 4 days PI, VEH-treated, infected mice exhibited significantly reduced corneal sensation at 6 days PI. In contrast, corneal sensation was significantly preserved in the AP20187-treated, infected group, as assessed with a Cochet-Bonnet esthesiometer (Fig. 4F).

### Depletion of CSF-1R^+^ Cells Correlates With Decreased Inflammatory Cytokine Protein Content and Blunted STAT3 Activation in the Infected Cornea

**Figure 5:**
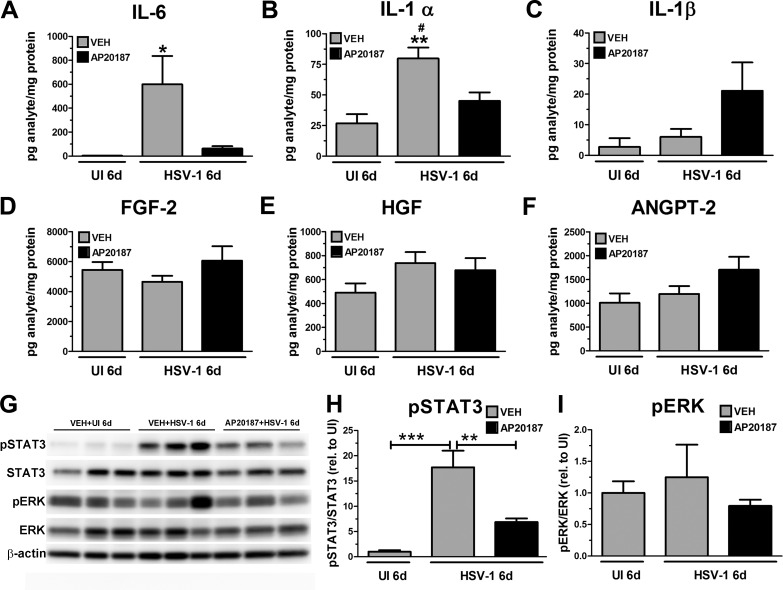
Depletion of CSF-1R^+^ cells correlates with decreased inflammatory cytokine protein content and blunted STAT3 activation in the infected cornea. MAFIA mice were treated with daily IP injections of AP20187 dimerizer or VEH. Following this treatment regimen, mice were infected with 10^3^ PFU HSV-1/cornea or left UI as controls, and their corneas harvested and processed for protein assays. (A–F) Cornea protein extracts were analyzed for soluble factor content by suspension array. Bars depict the mean pg/mg protein concentration for each analyte ± SEM (n = 4–7 for UI groups and n = 9–14 for infected groups per time point, from 3–4 independent experiments), ***P < 0.001 versus the rest of the groups in (A); **P < 0.01 versus VEH + UI and #P < 0.05 versus AP20187 + HSV-1 groups in (B) by ANOVA followed by Bonferroni multiple comparison test. (G) Representative immunoblot images from cornea protein extracts show VEH-treated, infected corneas have significant phosphorylation (activation) of STAT3 (pSTAT3) relative to UI controls, while AP20187-treated, infected corneas resulted in a significant reduction of pSTAT3. At 6 days PI, cornea protein extracts from mice that were pretreated with the VEH had significant elevation in pSTAT3 levels compared to the UI controls. Such phosphorylation was reduced in the AP20187 pretreated, infected cornea protein samples. Phosphorylation of ERK (pERK) was detected in UI and infected corneas, regardless of pretreatment. (H, I) Summary densitometry analyses of pSTAT3 and pERK. STAT3 and ERK detections were used to measure total STAT3 and ERK, respectively, and β-actin detection was used as a loading control. Internal normalization for each band was conducted relative to β-actin, and the results are expressed as ratios (H) pSTAT3/STAT3 and (I) pERK/ERK (relative to UI) ± SEM (n = 5–6 per group from two independent experiments). ***P < 0.001 and **P < 0.01 by ANOVA followed by Bonferroni multiple comparison test.

Proinflammatory cytokines driven by the transcription factor NF-κB are produced by epithelial infected cells, as well as inflammatory leukocytes in response to HSV-1 infection.^22,51,52^ We previously found in addition to preventing corneal nerve regression, the effect of treatment with the anti-inflammatory drug DEX prevented an elevation of NF-κB-driven cytokines IL-6, IFN-γ, and IL-1α in the cornea following infection.^18^ Furthermore, suppression of soluble IL-6 expression by neutralizing antibodies resulted in retention of corneal stromal and subbasal nerve networks and maintenance of corneal sensitivity through 8 days PI, which was interpreted as direct evidence of a role of inflammatory mediators in the mechanism of corneal nerve degeneration.^18^ To address whether the presence of macrophage infiltration into the infected tissue could be responsible for the up-regulation of inflammatory factors detected in the cornea undergoing nerve damage,^18^ we conducted assays on cornea protein extracts of MAFIA mice subjected to the different treatments. We found that in comparison to the UI controls, infected corneas of VEH-treated mice had significantly higher concentrations of IL-6 and IL-1α recovered 6 days PI (Figs. 5A, 5B). Such increases following infection were significantly reduced by AP20187 treatment. Regarding IL-1β, no significant differences were found between groups (Figs. 5A–C) (note: no IFN-γ in the cornea was detected at this time point under any of the treatments; data not shown). Unlike what was observed for inflammatory cytokine content, soluble proangiogenic factors assayed (FGF-2, HGF, and ANGPT-2) were constitutively expressed in the UI corneas and did not change as a result of VEH or AP20187 treatment following infection (Figs. 5D–F).

IL-6 signals through its membrane-bound or soluble IL-6 receptor (R) leading to the activation and dimerization of the signal transduction glycoprotein 130 (gp130).^53,54^ Through classic and trans-signaling pathways found in the cornea during inflammation,^55^ IL-6 signals via the tyrosine-protein kinase Janus kinase-2 (JAK2) and STAT3 to modulate the transcription of genes linked to the cell cycle, inflammation, apoptosis, cytokine signaling, and lipid metabolism.^56^ Despite failing to detect the phosphorylated form of STAT3 (pSTAT3), one study reported STAT3 was increased in the cornea via IL-6 stimulus in an experimental model of angiogenesis.^57^ To assess whether the increased presence of macrophages in the infected cornea was linked to increased activation of the downstream effector of IL-6, STAT3, we conducted Western blot analysis of pSTAT3 and total STAT3 (STAT3) in corneal protein extracts at 6 days PI. Compared to UI samples, corneas of MAFIA mice treated with VEH had significantly increased pSTAT3 (Figs. 5G–H). AP20187 treatment resulted in significantly blunted pSTAT3 compared to the VEH-treated, infected corneas (Figs. 5G–H). We also analyzed the expression and activation of the ERK1/2 MAPK signaling pathway reported to be expressed and phosphorylated in the cornea in response to inflammation associated with experimentally induced dry eye.^58^ Unlike STAT3, we found that ERK1/2 was constitutively phosphorylated in the corneas of UI mice, without significant changes upon infection, regardless of treatment (Figs. 5G–H).

### Effect of CSF-1R^+^ Cell Depletion on IL-6 Protein Content and STAT3 Activation in Infected TG

**Figure 6:**
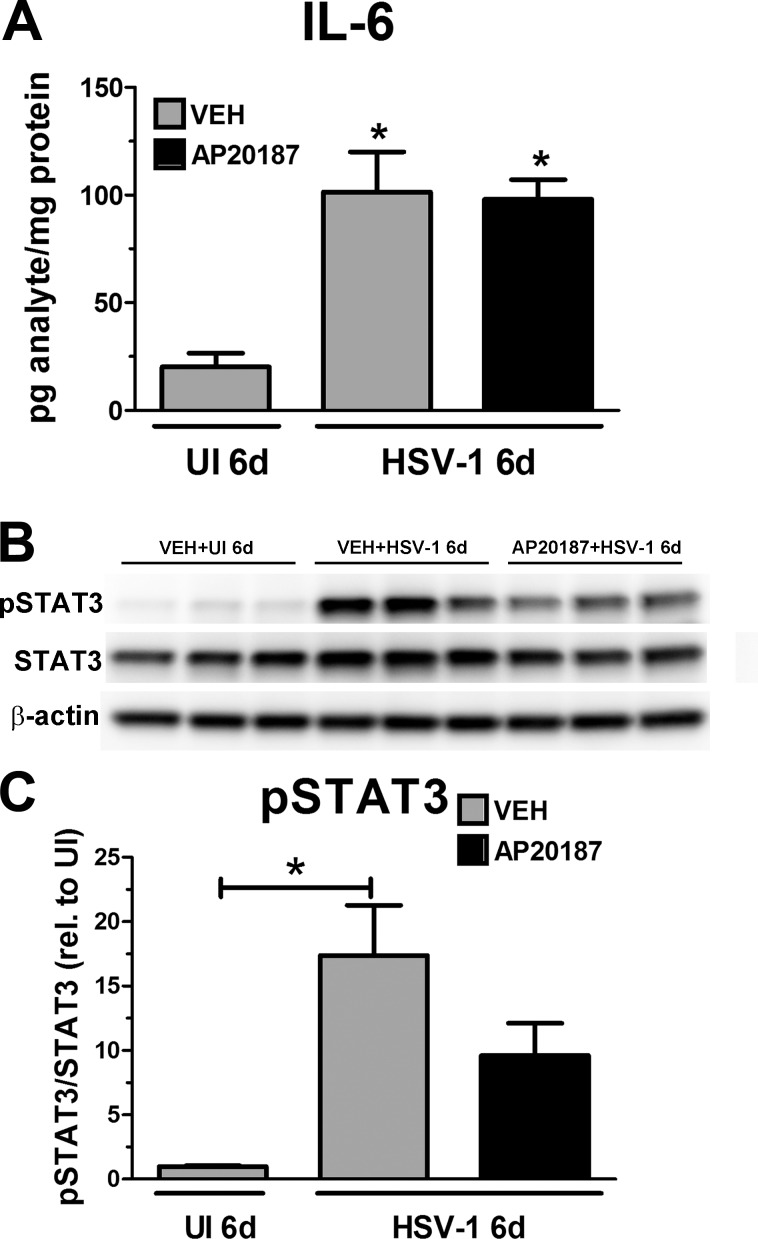
Effect of CSF-1R^+^ cell depletion on IL-6 protein content and STAT3 activation in infected TG. MAFIA mice were treated with daily IP injections of AP20187 dimerizer or VEH. Following this treatment regimen, mice were infected with 10^3^ PFU HSV-1/cornea or left UI as controls and the TGs harvested and processed for protein assays. (A) TG protein extracts were analyzed for IL-6 content by suspension array. Bars depict the mean pg/mg protein concentration of IL-6 ± SEM (n = 4 per UI group and n = 8–9 per infected group, from two independent experiments), *P < 0.05 versus the UI group by ANOVA followed by Bonferroni multiple comparison test. (B) Representative blot images from TG protein extracts showed both infected groups had increased phosphorylation (activation) of STAT3 (pSTAT3) relative to the UI control group at 6 days PI. (C) Summary densitometry analysis of pSTAT3. STAT3 was used to measure total STAT3 and β-actin detection was used as a loading control. Internal normalization for each band was conducted relative to β-actin, and the results are expressed as ratios (H) pSTAT3/STAT3 and (I) pERK/ERK (relative to UI) ± SEM (n = 5–6 per group from two independent experiments). ***P < 0.001 and **P < 0.01 by ANOVA followed by Bonferroni multiple comparison test.

Upon corneal inoculation with HSV-1, the virus replicates in the corneal epithelial cells and is retrograde transported through the sensory axons innervating the cornea to gain access to the soma of sensory neurons in the TG where viral replication and establishment of latency take place.^59^ We reasoned it is possible that aside from a local response in the infected cornea, inflammatory mediators produced in the infected TG, such as IL-6, activate signaling pathways that contribute to the corneal nerve regression.^60–62^ In order to address whether the AP20187 treatment affected IL-6 and pSTAT3 levels within the TG, we analyzed TG protein extracts from mice subjected to the VEH/AP20187 IP treatment. We found that in response to ocular infection, the concentration of IL-6 in the TG was significantly increased with no significant difference between AP20187 and VEH treatments (Fig. 6A). Western blot analysis showed a significant increase in pSTAT3 in response to infection with a trend toward a decrease following AP20187 treatment (Figs. 6B, 6C). We interpret these results to suggest that in the infected cornea undergoing nerve damage, IL-6 and STAT3 comprise a signaling axis modulated by the presence of CSF-1R^+^ macrophage infiltration, whereas in the TG, the activation of STAT3 is likely regulated by different or additional mediators not necessarily associated with the process of corneal nerve regression.

### Immunosuppressive Therapy Results in Decreased Activation of the STAT3 Signaling Pathway in the Infected Cornea

**Figure 7:**
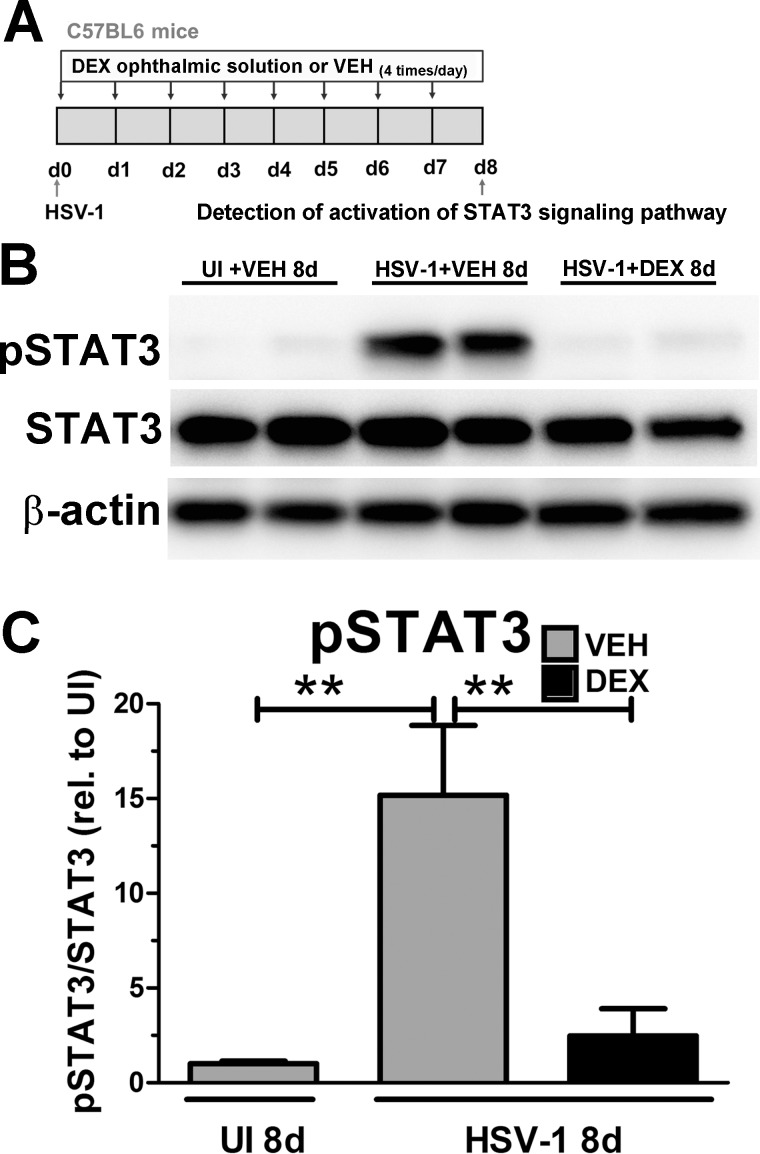
Immunosuppressive therapy results in decreased activation of the STAT3 signaling pathway in the infected cornea. WT mice were infected with 10^3^ PFU HSV-1/cornea or left UI as controls. (A) Starting at 2 hours PI, mice were topically treated with DEX or VEH (artificial tears) onto the corneas for 8 days PI prior to tissue collection and processing for Western blot analysis. (B) Representative blot images from cornea protein extracts showed infected, VEH- treated corneas had significant phosphorylation (activation) of STAT3 (pSTAT3) relative to UI controls, while infected, DEX-treated corneas had significantly reduced pSTAT3 levels. (C) Summary of the densitometry analysis of pSTAT3. STAT3 detection was used to measure total STAT3 and β-actin detection was used as a loading control. Internal normalization for each band was conducted relative to β-actin, and the results are expressed as ratio pSTAT3/STAT3 (n = 5–6 per group from two independent experiments), **P < 0.01 by ANOVA followed by Bonferroni multiple comparison test.

To address whether the signaling axis IL-6/STAT3 could be relevant in a different model of immunosuppression, we used a model that incorporated DEX administration on the corneal nerve network of WT mice infected with HSV-1.^18,19^ In this model, the elevation of IL-6 protein content in the infected WT cornea was completely suppressed by repeated daily treatments of the cornea with DEX consistent with significant preservation of corneal nerve structure and sensitivity by 8 days PI.^18^ As a follow up, in the present study Western blot analysis of cornea protein extracts of mice treated with artificial tears (VEH) on the corneas during the first 8 days after infection showed a significant increase in the pSTAT3 levels compared to UI controls (Fig. 7). Topical DEX treatment (Fig. 7A) completely suppressed the pSTAT3 (Figs. 7B, 7C) consistent with its ability to completely suppress IL-6 up-regulation in the infected cornea.^18^ These results further confirmed the activation of the STAT3 signaling pathway in the context of HSV-1 triggered inflammation, and such activation is suppressed upon immunosuppressive therapy. In conclusion, we have demonstrated that two different models that cause significant reduction of macrophage infiltration in the cornea, and significant reduction in the content and biological activity of soluble inflammatory mediators upon infection, have a common effect in preventing corneal nerve damage.

## Discussion

In the cornea, HSV-1 infection elicits a response by resident cells, including infected epithelial cells, that release chemokines that recruit leukocytes in two successive waves consisting of polymorphonuclear cells (PMNs), early responding DCs, macrophages, inflammatory monocytes, and natural killer (NK) cells followed mainly by CD4^+^ and CD8^+^ T cells.^11,51,63,64^ Recent reports have partially addressed the relationship between specific immune cells and corneal nerves in regard to HSV-1 infection. The normal population of resident cDCs was suggested as required to preserve corneal nerves during the acute phase of HSV-1 infection.^19^ In addition, CD4^+^T cells were shown to influence the long-term persistence of nerve defects at time points consistent with viral clearance and establishment of latent infection.^22^ The present study adds novel insights into the mechanism of corneal nerve regression following HSV-1 infection. We propose myeloid-lineage cells infiltrating the cornea tissue in response to infection are part of a local response that triggers an elevation of inflammatory cytokines and downstream activation of the gp130/STAT3 pathway to mediate corneal nerve degeneration. Our findings are clinically relevant as they might improve our understanding of the molecular events of virus-induced NTK, which could be common to peripheral neuropathies of different etiologies that develop as a result of inflammation.

Although we recognize the transgenic MAFIA mouse model used in the present study is specific for the CSF-1R^+^ lineage (CD45^+^CD11b^+^GFP^+^ population composed by monocytes, macrophages, and some cDCs) and no single cell-specific population, it is a valuable tool to uncover a potentially relevant pathway controlling HSV-1 induced nerve regression driven by the elicited innate immune response to infection. A consideration taken into account in our experimental design was the reported reversible nature of cell depletion by the dimerizer AP20187 in the MAFIA system.^36^ The observation that the CSF-1R^+^ cells were found to repopulate after 1 week of cessation of the treatment was used to select an end point of analysis of 6 days PI to avoid confounding results. Of note, 6 days PI is a time point consistent with physical and functional loss of nerves that is further advanced by 8 days PI.^17,18^ The influx of adaptive immune cells (CD4^+^ and CD8^+^ T cells) in the cornea was significantly increased by 8 to 10 days of HSV-1 infection.^18,45^ Given the end point of the present study is 6 days PI, it is possible that cells other than macrophages and their products, have a role in the progression/late stages of nerve degeneration.

The successful depletion of monocytes/macrophages measured in blood and lymphoid tissues but not in the cornea prior to infection set an advantage in the interpretation of our results. Specifically, by maintaining the resident macrophages/cDCs, the dimerizer treatment allowed for an association between the observed phenotype of nerve protection with a reduced corneal influx of CSF-1R^+^ cells, mainly derived from the circulation in response to HSV-1 infection. In agreement with the hypothesis that nerve degeneration is driven by the elicited immune response within the cornea, and not local HSV-1 replication,^18^ the viral content in the infected corneas treated with AP20187 was similar to the control group. Not surprising and likely due to the implication of macrophages in limiting dissemination of HSV-1 infection in the peripheral nervous system^64^ and coordinating the CD8^+^T cell response crucial for viral clearance and establishment of latency,^65^ the viral titer in TG of AP20187-treated mice was significantly elevated compared to the VEH-control group. We interpret these results to suggest HSV-1 infection in sensory neurons in the TG has no clear role in the degeneration of corneal nerves in our model within the confines of the timeframe under study.

By using different models of immune suppression, (1) the MAFIA mouse system and (2) topical DEX therapy, the study identifies a potentially relevant signaling axis for the regression of corneal nerves. In both models, there was a clear correlation between increased IL-6 expression and activation of the downstream signaling transcription factor STAT3 with the loss of the corneal nerve network. We submit the up-regulation of IL-6 (along with other soluble signals) by resident cells (i.e., epithelial cells, keratocytes, and resident immune cells) and/or CSF-1R^+^ cells is responsible for the activation of gp130/STAT3 in the inflamed cornea as part of the mechanism that triggers the regression of corneal nerves. We speculate the deletion of STAT3 intrinsic to myeloid cells would help elucidate whether the signaling targets of IL-6 up-regulation in the milieu of the HSV-1 cornea are resident, hematopoietic-derived, or nonhematopoietic-derived corneal cells, infiltrating myeloid cells, or both.

We recently reported FGF-2 regulates the progression of corneal neovascularization beyond resolution of infection (14–21 days PI, latent infection) and its neutralization suppresses the expression of proangiogenic factors and preserves visual acuity without altering corneal sensation.^45^ Of note, in the present study we found FGF-2 was highly expressed in the UI cornea and its content not modified as a result of acute infection (6 days PI) or CSF-1R^+^ cell depletion. We speculate the basal high contents of FGF-2 might explain the constitutive phosphorylation of ERK1/2 observed in the cornea, independent of infection or cell depletion.

Data suggest the relevance of the IL-6/STAT3 axis in the cornea but not within the TG orchestrate the nerve retraction, since macrophage lineage depletion resulted in nerve protection and correlated with a decrease in IL-6 and pSTAT3 in the cornea but not the TG. Consistent with another report,^66^ we found ocular HSV-1 infection elicits an increase in IL-6 protein expression in the TG at 6 days PI. The elevation of IL-6 was sustained and correlated with decreased pSTAT3 following AP20187 treatment. Considering the loss of the “linear relationship” between IL-6 and STAT3 activation within the TG, and a higher susceptibility to TG infection as a result of cell depletion, it is conceivable that the biological effects of IL-6 and STAT3 in the infected TG are associated with other functions other than nerve retraction, such as cell survival/repair and viral clearance in the nervous system.^67–69^

We submit the loss of nerves in response to local HSV-1 infection is orchestrated, at least in part, by the elicited innate immune cells (likely macrophages and/or inflammatory monocytes) that infiltrate the cornea. We speculate such immune cells directly or indirectly trigger a local response, comprising inflammatory cytokines including IL-6 and downstream activation of the gp130/STAT3 pathway to mediate nerve retraction/damage. Future studies using models of gain and/or loss of function are required to further define the direct role of the STAT3 pathway in corneal nerve degeneration.
